# Structural Insights into tRNA Dynamics on the Ribosome

**DOI:** 10.3390/ijms16059866

**Published:** 2015-04-30

**Authors:** Xabier Agirrezabala, Mikel Valle

**Affiliations:** Structural Biology Unit, CICbioGUNE Biscay Technology Park, Bld 800, 48610 Derio, Basque Country, Spain; E-Mail: xagirrezabala@cicbiogune.es

**Keywords:** ribosome, tRNA dynamics, protein synthesis, structure, conformational landscape

## Abstract

High-resolution structures at different stages, as well as biochemical, single molecule and computational approaches have highlighted the elasticity of tRNA molecules when bound to the ribosome. It is well acknowledged that the inherent structural flexibility of the tRNA lies at the heart of the protein synthesis process. Here, we review the recent advances and describe considerations that the conformational changes of the tRNA molecules offer about the mechanisms grounded in translation.

## 1. Introduction

The key component of protein synthesis is the ribosome, a macromolecular device formed by two subunits of distinct size composed of RNA (ribosomal RNA or rRNA) and proteins. This supramolecular structure mediates the steady addition of amino acids (aas), carried by the transfer RNAs (tRNAs), into the emerging protein chain via peptide bond formation. By virtue of the idiosyncratic arrangement of the small and large subunits, a ~100 Angstroms (Å) long chamber is formed in the inter-subunit space, which is used by the tRNAs to cross the entire ribosome in a coordinated manner as they bind the primary interaction slots called A (for aminoacyl), P (for peptidyl) and E (for exit) sites [1]. In this scenario, the tRNAs play as dynamic agents that facilitate the synergism among the small and large subunits. Indeed, the main steps of protein translation, *i.e.*, initiation, aa-tRNA incorporation (decoding) and translocation are in essence coupled to the structure and dynamic behavior of tRNA molecules.

**Figure 1:**
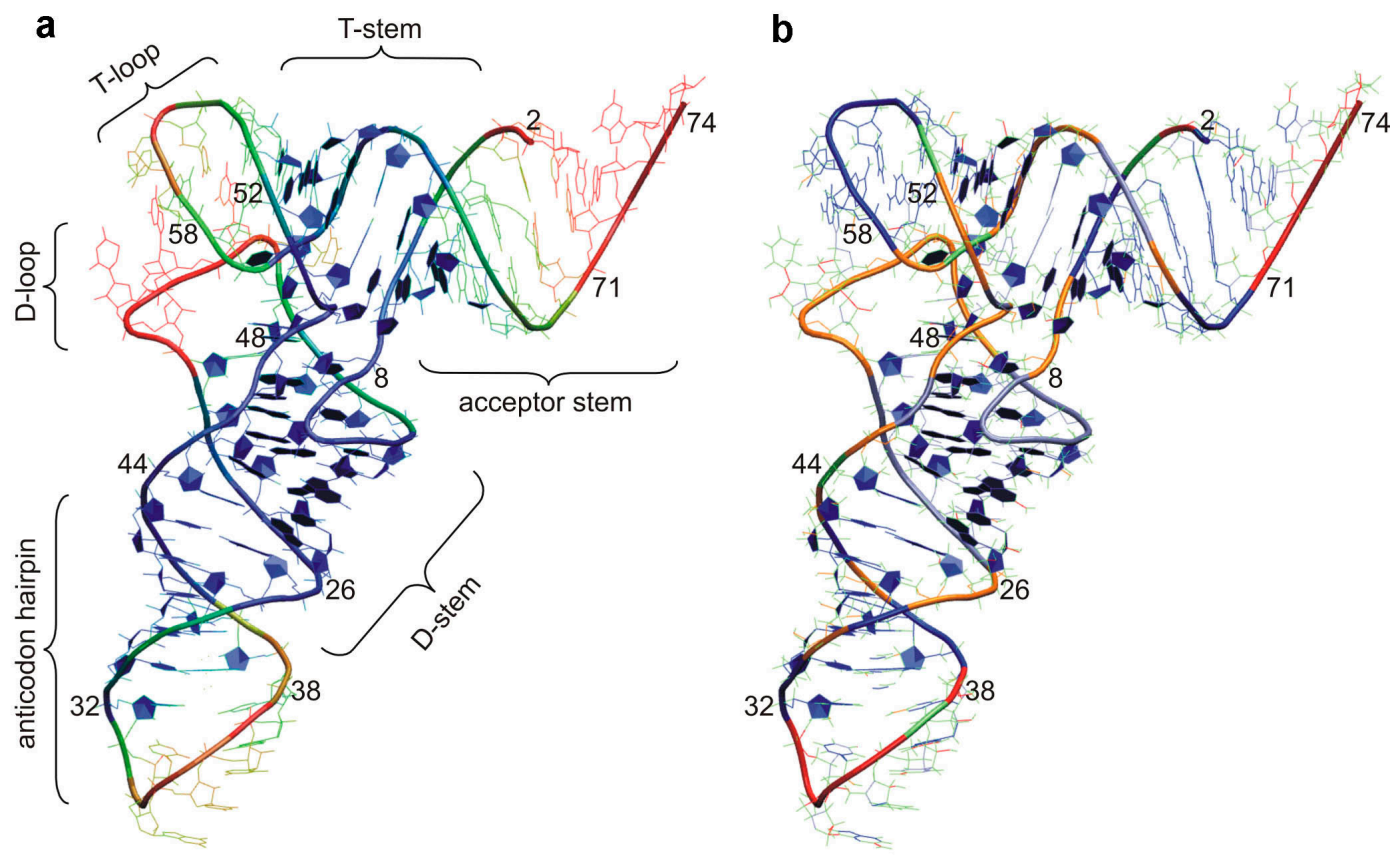
Tertiary structure and computational analysis of tRNA’s structural plasticity. (**a**) Color-coded B factor information, which can be described as flexibility estimation, is displayed for the phosphorus atoms of tRNA^Asp^ X-ray structure (PDB 2TRA) [8]; (**b**) Color-coded flexibility prediction by constraint counting for the same tRNA species, in which overconstrained regions are indicated in blue, rigid regions in green, and flexible regions in red. (Figure adapted from [9], Copyright 2008 Elsevier).

The tRNA secondary structure is commonly displayed in a cloverleaf shape [2], in which distinct stems are stabilized by Watson-Crick base pairings: the anticodon stem loop or ASL (complementary to the messenger RNA, mRNA’s codon), the D stem loop, the acceptor stem (which shows the conserved 3' terminal CCA sequence to which the aa has been linked by aminoacyl tRNA synthetases or aaRSs) and the T-arm, in addition to the extra loop (which is variable in size and not present in all tRNA species). Early X-ray structure determination of yeast tRNA^Phe^ showed that tRNAs form a compact l-shaped molecule [3,4,5,6]. In this l-shaped tertiary configuration, one extreme is formed by the acceptor stem and T-arm, whereas the distal part is formed by the D and anticodon stems (Figure 1a). As anticipated by Crick [7], tRNA shape is directly correlated with its workings as the adaptor molecule during translation: The anticodon at one end reads the mRNA; the acceptor stem at the other end carries the cognate amino acid.

**Figure 2:**
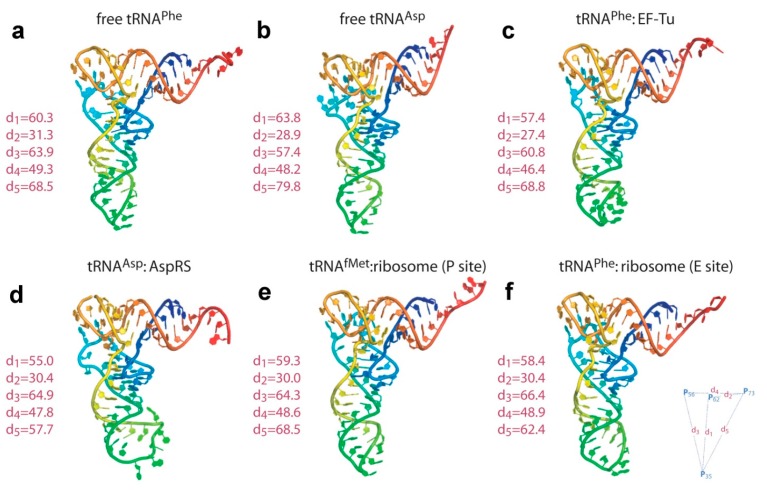
A gallery of tRNA atomic structures, isolated or in complex with EF-Tu, aminoacyl tRNA synthetase (RS) or the ribosome. (**a**) PDB 4TRA [8]; (**b**) PDB 2TRA [8]. (**c**) PDB 1TTT [11]; (**d**) PDB 1ASY [12]; (**e**,**f**) PDB 4V51 [13]. The different regions of the tertiary structure of tRNA are color-coded. Variability is mainly manifested on the distal parts (*i.e.*, ASL and CCA end), as well as in the relative angle between the anticodon and acceptor stems. Plasticity is also readily observed in the D- and T-loop regions. Measured distances between some phosphorus atoms (in Å) are shown on the left side of the structures. (Figure adapted from [14], Copyright 2008 Nature Publishing Group).

Since the first crystallographic structures, a wealth of data has shown that tRNA molecules display a common global shape, the main difference between them being the angle formed by the two arms of the l-shaped structure (Figure 2). The increasing number of structures has also revealed variability in structural details to specifically interact with the corresponding maturation/modification enzymes or aaRSs. This is especially true in the case of some mitochondrial tRNAs for instance, which display unique structural features. Details notwithstanding, the prevalence of an identical architectural framework in all tRNAs, either canonical or divergent, in all three kingdoms of life is undeniable (for an excellent review, see [10] and references therein). At the same time, tRNA molecules not only traverse the inter-subunit space during protein synthesis, but can also rearrange their l-shaped structure as the distortions of the tRNA molecule during its course of action are significant. Indeed, it is acknowledged that these reconfigurations are imperative during many stages of the protein translation elongation step.

In this review, we will first summarize the contributions made by experimental (mainly kinetic and smFRET studies) and computational methods to our understanding of the variations in the structure of the tRNA when bound to the ribosome, giving a sense of its flexible nature. We will then turn to recent, exciting discoveries related to the variability of the tRNA structure as visualized by cryoEM (cryo-electron microscopy) or X-ray crystallography during initiation, decoding and translocation (*i.e.*, the elongation cycle of protein synthesis). After analyzing the implications of such snapshots on the protein synthesis process, the review will conclude with an account of a recently described tRNA configuration not directly related to protein synthesis but to a metabolic sensing activity.

## 2. tRNA Dynamics an Overview

The potential flexible nature of the tRNA architecture was already acknowledged on the basis of the very first atomic structures. Considering the l-shape of the tRNAs, which are formed via weakly interconnected double helices (formed by the anticodon stem-D stem, and the acceptor stem-T stem, respectively), the flexibility of the tRNA molecule was assumed to be based on hinge-bending-type reconfigurations. However, experimental support for the existence of structural plasticity in the ribosome-bound forms did not come until chemical protection experiments showed that the binding of the incoming aa-tRNA in the ribosomal A site occurs in several steps via the formation of a so-called A/T state of binding [15]. In this conformation, the acceptor 3' end is still bound to elongation factor Tu (EF-Tu), whereas the ASL interacts with the mRNA. Along these lines, and confirming a previous hypothesis based on the existence of intermediate states in the movement of tRNAs through the ribosome [16], it was also shown that after the peptide bond formation, the acceptor ends of the A- and P-site tRNAs spontaneously reconfigure to form the so-called tRNA hybrid states [17]. In this configuration, the ASL of the tRNAs locate in the 30S A and P sites, while the acceptor stems are oriented towards the 50S P and E sites.

Subsequently, kinetic [18] and single molecule FRET (Förster resonance energy transfer) [19] experiments have showed in increasing depth and detail that the stepwise incorporation of the aa-tRNA into the A site of the ribosome is associated with the formation of discrete structural intermediates such as the A/T state, which is characterized by a change in the conformation of the D-loop [20,21,22,23,24,25,26,27]. Additional experiments have also provided evidence about the role of the formation of the hybrid state of tRNA binding for the translocation process, the rapid dynamic exchange between both states, or have highlighted the interplay of the tRNA molecules with elongation factors or other ribosomal parts/motions involved among other relevant features [28,29,30,31,32,33,34,35,36,37,38,39,40,41,42,43,44].

In the meantime, conformational changes in the tRNA molecules have been extensively studied by computational methods, providing an extra link between structure and dynamics. Approaches such as normal mode analyses (NMA) or molecular dynamic (MD) simulations among others have allowed deeper insights into the dynamics of tRNA structures at atomic detail. Insight into tRNA flexibility was initially obtained from analysis of non ribosome-bound tRNA structures because of experimental and/or computational limitations (e.g., [9,45,46,47,48,49,50,51,52,53]); these studies nonetheless were able to capture the D-loop and 3' CCA end’s flexibility or the inter-arm angle dynamics among other relevant features (Figure 1b).

The progress of computing power and capability has subsequently allowed to perform all atom, longer time simulations, as well as the simulation of tRNA’s structure dynamics in complex with its various binding partners, for instance aaRS-s [54] or elongation factor EF-Tu [55]. Most relevantly, the wealth of structural data for ribosomal complexes coming from X-ray in the last decade (specific examples will be shown in the next section), have allowed to dissect the conformational space sampled by the tRNA molecules during multiple stages in the context of the entire ribosome, as well as to propose the structural and dynamic features of short-lived transition states [56,57,58,59,60,61,62,63,64]. These analyses have been a great complement to structural and dynamic fluorescence studies [65,66].

Based on the data accumulated over years of research, the plasticity of the tRNA molecules at different stages of protein translation has been finally shown to be relevant to the whole process. All in all, these data led to the suggestion of the tRNA behaving like a molecular spring that interchanges relaxed, low energy and higher energy configurations [67,68].

## 3. Structural Snapshots of tRNA Conformation Plasticity during Translation

### 3.1. Initiation

Briefly, during translation initiation in prokaryotes, the 30S small subunit binds to the initiator tRNA (fMet-tRNA^fMet^), initiation factors (IF1, IF2 and IF3) and mRNA to form the 30S pre-initiation complex in a multi-step process. Once the mRNA is correctly positioned, and the anticodon of the initiator tRNA is base-paired with the initiation AUG codon of the mRNA, sequential release of the factors (mediated by IF2’s GTP hydrolysis) promotes the association of the 50S large subunit to form the initiation 70S complex [69,70,71,72,73,74,75].

CryoEM analysis of initiation 70S complexes has shown that the initiator tRNA is held in a characteristic position by two interactions: One involving the anticodon stem which is hidden in the P site of the small subunit, and the other between IF2 and the tRNA acceptor stem [76,77]. The orientation of initiator tRNA differs from the canonical P site position as the *C*-terminal domain of IF2 sterically precludes a “classical” P site binding of the initiator tRNA molecule, and instead, forces the tRNA to acquire an intermediate configuration (termed P/I) between the classical P and hybrid P/E sites [76]. The structures also suggested a ~20° rotation of the ASL, which displaces that CCA end by 28 Å towards the E site. On the contrary, more recent cryoEM analysis of 30S initiation complexes showed that the CCA end of the tRNA was instead oriented towards the A site [78]. This new state, called P/I1, showed a rotation of ~15° of the acceptor stem. A somewhat different P/I state was also reported for the 30S IC from *T. thermophilus* in the absence of IF3 [79], which may be related to IF3’s role in the stability of tRNA binding to the initiation complex.

The eukaryotic initiation of translation, a highly regulated stage, is a very complex process as it involves 11–13 eIFs. Whereas procaryotes can use the SD (Shine-Dalgarno) sequence present in more than 40% of all bacterial genes to locate the start codon [73,80,81,82], eukaryotic ribosomes use a scanning mechanism [83]. During the initial stage of initiation, the 40S small subunit, supervised by initiation factors eIF1, eIF1A and eIF3 binds to the ternary complex (TC) formed by eIF2, GTP and initiator tRNA. The subsequent cap-dependent interaction of this 43S complex with the 5' end of the mRNA is guided by another factor, eIF4E, as well as eIF4Ga and polyA binding protein (PABP), that interact with the 3' end. The next step, in which initiation factors eIF4A, eIF2 and eIF5 play a role, involves the formation of the 48S complex (at this point, the initiator tRNA is already base-paired to the start codon). The next stages in the formation of the 80S initiation ribosome complex entangle the binding to the 60S large subunit, hydrolysis of GTP by eIF5B and subsequent release of the factors to correctly place the initiator tRNA in its final position [83,84,85,86,87,88].

**Figure 3:**
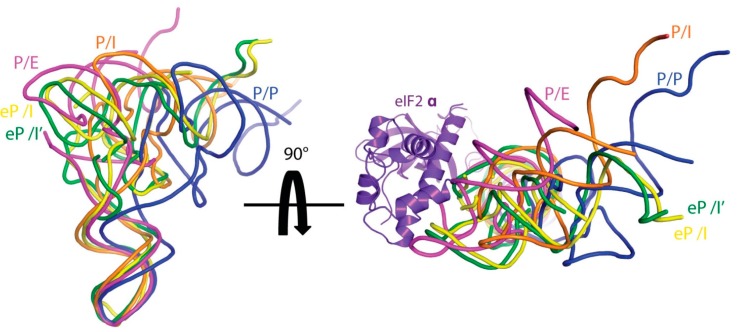
Comparison of tRNA structures from different eukaryotic initiation complexes. The shown tRNA structures are the following: eP/I’ (PDB 3J81 [97]), eP/I (archaeal Met-tRNAiMet [102] fitted as rigid body in EMD 5658 [95]) and P/I (PDB 4KZZ [94]), which are compared to classical P/P (PDB 4V51 [13]) and hybrid P/E (PDB 4V9H [103]) tRNAs. In the view at the right side, eIF2a is also shown. (Figure adapted from [97], Copyright 2014 Elsevier).

Initial low resolution studies (e.g., [89,90]) have led the way to higher resolution studies that have provided relevant snapshots of the eukaryotic initiation process. These structures include complexes formed by 40S∙eIF1 [91], 40S-eIF1-eIF1A [92] and 60S-eIF6 [93] from *Tetrahymena thermophila*, mamalian 40S-eIF1-eIF1A [94] and 43S complex formed by eIF3, TC and Dhx29 (an RNA helicase), but without mRNA, eIF1, eIF1A or eIF5 [95], as well as yeast *S. cerevisiae* 40S-eIF1-eIF3 [96] and *Kluyveromyces lactis* 48S complexes containing eIF1, eIF1A, mRNA, and the TC complex formed by eIF2, GTP and initiator tRNA [97]. These structures show that the 40S binding site for eIF1 sterically impedes the standard P site binding of the initiator tRNA molecule, as it happens in prokaryotes. The 48S complex for instance [97], displays the initiator tRNA (eP/I’ in Figure 3), once base paired to the start codon of the mRNA after the initial scanning (in a conformation called PIN state, see [98]), as opposed to the POUT initiator tRNA described by Hashem and coworkers [95] in the absence of mRNA. In this PIN configuration, the CCA end shows an upward shift due to steric clashes with eIF2, and the acceptor stem and T loop are directed toward the A and E sites, respectively. Considering the acceptor stem position, the reconstructions indicate that the PIN state initiator tRNA in eukaryotes is similar to the prokaryotic P/I configuration [76,79], but not to the P/I1 state [78]*.* Also worth mentioning is the yeast 80S ribosome in complex with Met-tRNA_i_^Met^, mRNA and eIF5B [99,100], which mimics the last stage of the initiation complex formation. The structure shows how eIF5B stabilizes the initiator tRNA in a conformation that restricts the 3' end from reaching the PTC (peptidyl transferase center), and thus, blocks the formation of the final 80S initiation (*i.e.*, elongation competent) complex until hydrolysis of GTP and release of eIF5B, in a similar fashion to its bacterial ortholog IF2 [101].

Altogether, the diversity of states for initiator tRNAs (Figure 3 show some examples described in eukaryotes) reflect the flexibility of the acceptor arm and that the distinct observed positions sampled during the first steps of translation might regulate the assembly of ribosomal subunits and the incorporation of the initiator tRNA into the final P site [78].

### 3.2. Decoding

For the incorporation of the correct aa-tRNA, the ribosome has to distinguish cognate ternary complexes (formed by aa-tRNA^aa^·EF-Tu·GTP in prokaryotes) from the near-cognate and non-cognate species. The decoding process is based on a kinetic discrimination mechanism, and can be divided into two stages, GTPase activation and accommodation, which are separated by the irreversible hydrolysis of GTP by EF-Tu [104,105,106,107,108,109]. This approach, based on a kinetic proofreading mechanism, grants multiple chances to reject the incorrect tRNA species [110]. Structurally, decoding relies on the mRNA codon and the tRNA anticodon’s Watson-Crick complementarily, which is monitored by nucleotides G530, A1492 and A1493 from 16S rRNA [111,112], as well as 30S conformational changes upon cognate codon-anticodon match [113], although it should be noted that some controversy raised on this subject recently [114,115,116]. All in all, however, it is clear that the ribosome has an active role in the accuracy of the whole process, using induced fit mechanisms (a combination of local and global conformational changes) to exclusively accelerate the forward rates of GTPase activation and accommodation for cognate species and thus, control the fidelity of tRNA selection (for more details, see [115,117,118,119,120] and references therein).

**Figure 4:**
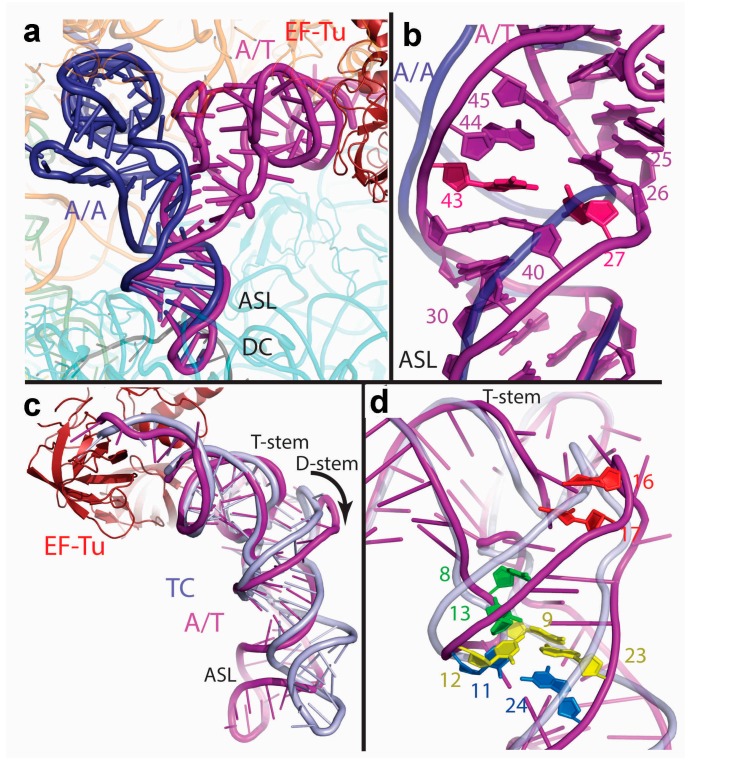
The A/T state of tRNA binding. (**a**) Comparison of the A/T tRNA (PDB 4V5G [129]) with the A/A tRNA that mimics the accommodated state (PDB 4V5D [133]); (**b**) Major changes are observed in the ASL region, as the helical twist is reduced after base pair 30:40. Also, the helical strands split at nucleotides 25 to 45 and 26 to 44. The well-known 27:43 base pair mutation (highlighted in pink) results in an error-prone phenotype [134], as it probably weakens the ASL stem and thus, facilitates the strand separation that leads to the final distorted form of the incoming tRNA; (**c**) Comparison of the A/T tRNA (PDB 4V5G [129]) with the EF-Tu bound form (TC) in the absence of the ribosome (PDB 1TTT [11]); (**d**) A gallery of mutations/manipulations in the tRNA molecule that affect the accuracy of decoding are highlighted: Cross-linking of nucleotides 8 and 13 [135], and mutations at 9:12:23 [136] and 24:11 [136,137,138]. Note that contrary to previous assumptions, subsequent structural analysis by Schmeing and coworkers [130] discarded the idea that the so-called Hirsh mutation influences the flexibility/deformability of the tRNA. This framework also puts into context previous kinetic experiments: see proflavin insertions at positions 16 and 17, e.g., see [20]. (Figure adapted from [129], Copyright 2009 Am. Assoc. Adv. Sci.)*.*

**Figure 5:**
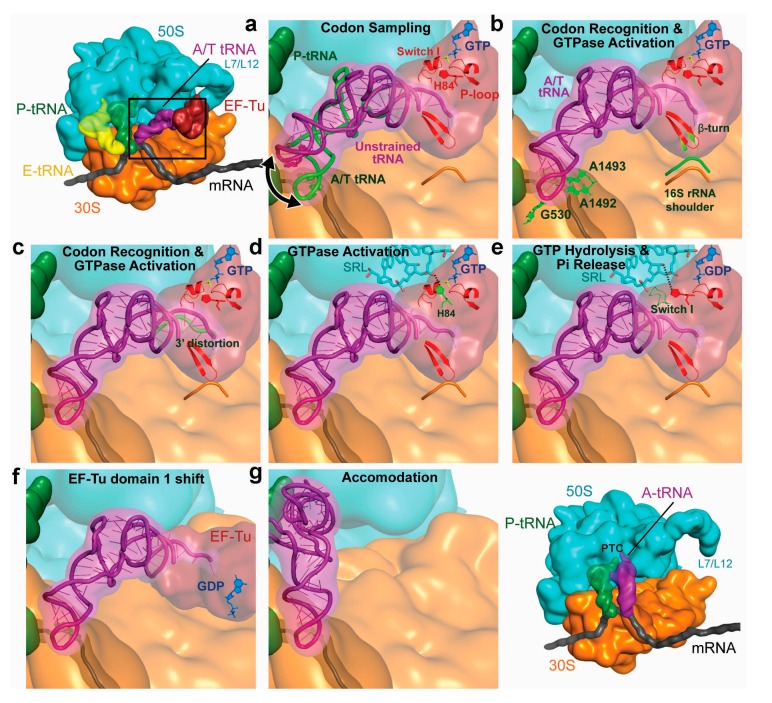
Graphic representation of the sequential stages during tRNA incorporation. (**a**) Distortion of the aa-tRNA during the initial, reversible codon sampling stage; (**b**) After codon recognition, the tRNA is stabilized in an A/T form. The distorted A/T state is known to be critical for the subsequent activation of GTP hydrolysis on the ribosome [138,139]. Accompanying global changes of the 30S subunit (mostly comprising the shoulder region in 16S rRNA) lead to the conformational change of domain II’s β loop; (**c**) which causes the distortion of the acceptor end; (**d**) Concomitant disruption of the contacts with switch I region of EF-Tu relocate the catalytic residue His84 [140] into the proper orientation. A2662 of the sarcin-ricin loop (SRL) of the 23S rRNA is also involved. Note that some details about the GTPase activation mechanism are still being discussed (see [131,141,142] for more details); Release of Pi is coupled to the (**e**) disordering of the switch I loop and (**f**) subsequent conformational change of EF-Tu [143]; (**g**) Finally, EF-Tu is released and the tRNA molecule relaxes its conformation to accommodate in the vacant A/A site. All in all, the different decoding stages are governed by the dynamic nature of the tRNA molecule within the A site [27]. Note that as the sequence and structure of each aa-tRNA is tuned according to the nature of the amino acid they carry, as well as to the codon-anticodon strength, the different cognate tRNA species display unvarying decoding properties (*i.e.*, uniform rates of acceptance by the ribosome) [144,145]. (Figure adapted from [115], Copyright 2013 Annual Reviews).

Distinct transient states in the pathway of aa-tRNA delivery to the A site have been described, initially by kinetic methods that follow the fluorescence signal of strategically placed proflavin molecules [20] (see Figure 4d), later also via smFRET [26,27], showing fluorescence changes in the anticodon region and the D loop of tRNA along the pathway. Essential structural information into the aa-tRNA incorporation process has been obtained through the analysis of one of such intermediates [15]. This state has been analyzed by cryoEM in ribosomal complexes bound with different ternary complexes in the presence of the antibiotic kirromycin [121,122,123,124,125,126,127,128], as well as X-ray [129,130] (Figure 4). When bound to kirromycin, elongation factor EF-Tu mimics the GTPase-activated configuration, albeit in a post-hydrolysis scenario [20]. The crystal structure of the ternary complex bound to the ribosome along with the antibiotic paromomycin and GTP analog GDPCP on the other hand, has suggested how GTPase activation might occur on the ribosome [131]. In all these structures, the aa-tRNA molecule adopts a distorted configuration, the distortion being located between the anticodon- and D-stem loop regions. The structures show that the tRNA deformation and the resulting spatial relationship with EF-Tu and the ribosome is responsible for the correct cognate codon recognition that activates the factor itself for GTP hydrolysis (see Figure 5 and corresponding legend for more details). Computational simulations have suggested that a corridor formed by conserved residues is responsible for directing the CCA end toward the PTC during accommodation [57], and that the process relies on reversible structural fluctuations of the accommodating aa-tRNA [60], very much in the same vein as during initial selection [132].

**Figure 6:**
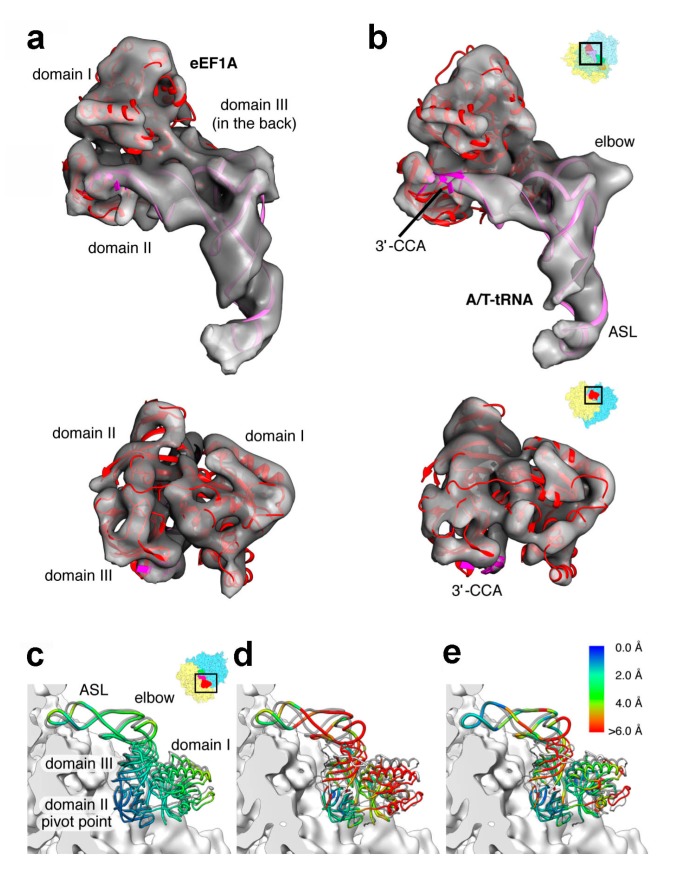
Comparison of mammalian and bacterial ternary complexes. CryoEM maps of eukaryotic (**a**) codon sampling and (**b**) GTPase activation steps. Fitted archaeal aEF1α (PDB 3VMF [148]) and A/T tRNAs (ASL part from PDB 4V5L [131], body from PDB 1TTT [11]) are shown in red and pink ribbons, respectively; (**c**) Superposition of codon sampling (grey) and GTPase activation state ternary complexes after alignment of 40S subunits. Superposition of bacterial GDPCP stalled ternary complex, in grey (PDB 4V5L [131]) with eukaryotic (**d**) codon sampling and (**e**) GTPase activation state ternary complexes after alignment of 18S/16S rRNA’s conserved parts (the electron density corresponding to the 40S subunit surface is shown in white); (**e**) The relative orientations of factor and tRNA diverge in the two states of the eukaryotic decoding complex, as well as in their bacterial counterpart, which leads to a different interaction mode with the ribosome. The largest differences are observed in the tRNA elbow region due to interactions with the SRL and H89 from the large subunit, interactions not observed in bacteria. Measured distances (in Å) between the ternary complexes shown in panels (**d**,**e**) are color coded and shown on the right side of panel. (Figure adapted from [146], Copyright 2014 Elsevier).

Recent cryoEM structures of mammalian decoding complexes in the presence of GMPPNP have shown that as it is the case in bacteria, the key events in decoding are also the interactions of the ASL with the decoding center (A1824/A1825 of 18S rRNA) and the signaling of this event to the GTPase center of EF-Tu’s eukaryotic counterpart eEF1A [146]. The data also included structural information on the codon sampling conformation prior to codon recognition that is lacking in bacteria (Figure 6). Both A/T substates (initial recognition and GTPase activated) were differentiated due to the configuration of the decoding center, as well as eEF1A’s interaction pattern with the universally conserved sarcin ricin loop/SRL, which is an essential part of the factor binding and activating site (H95 of 28S rRNA) [147]. The ASL was visualized buried deeper in the decoding center once the codon was recognized. Conformational changes in the shoulder and head regions of the 40S subunit after the stabilization of the codon-anticodon duplex, similar to those described in the case of the bacterial domain closure rearrangement [113] were also detected. Nonetheless, the tRNA was visualized in the distorted configuration in both stages.

When these mammalian structures were compared to the bacterial kirromycin [129] or GDPCP [131] stalled complexes, differences were observed in the relative position of ribosomal parts that interact with the ternary complex (Figure 6). Consequently, whereas the position of ASL and CCA end were found to be similar, differences in the tRNA elbow configuration were observed, leading to specific interactions with the large subunit in both codon sampling and GTPase activation states. Finally, the data also described for the first time a ~6 degree rotation of the 40S subunit toward the L1 stalk, perpendicular to the inter-subunit rotation observed during translocation (see next section) and termed subunit rolling. Apparently, this rearrangement predominantly occurs during the accommodation of the tRNA from the A/T to the A/A state. Details notwithstanding, it is clear that in both prokaryotic and eukaryotic systems, the physical properties of the tRNAs have constantly adapted to enable the whole decoding process in which structural distortion plays a fundamental role.

### 3.3. Translocation

**Figure 7:**
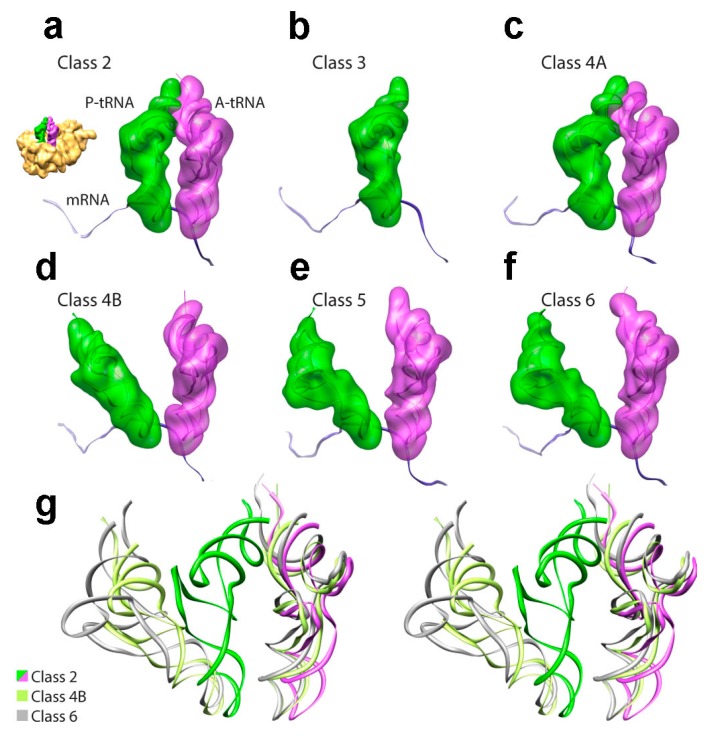
Comparison of translocation intermediates obtained by cryoEM and particle sorting. (**a**–**f**) Maximum likelihood methods were applied to a dataset of pre-translocational complexes in the absence of EF-G, obtaining an ensemble of substates that encompass a large conformational space. Out of the initial six classes, one subset (class 4) was further subdivided due to residual heterogeneity (class 1 is not shown as it was deemed to be artifactual due to bias in particle orientations). Class 2 (**a**) and class 4A (**c**) correspond to the classic A/A and P/P tRNA configuration, while class 3 (**b**) corresponds to ribosomes bearing a single tRNA configuration (P/P). Classes 5 (**e**) and 6 (**f**) are similar to each other, and to the previously characterized A/P and P/E hybrid configurations [160,161]. Class 4B (**d**) represents a new intermediate in which the P-site tRNA’s elbow is halfway through its transition from classic to hybrid state, a configuration that is coupled to an intermediate intersubunit rotation and L1 stalk movement; (**g**) Stereo-view of the superposition of A- and P-site tRNAs from class 2 (classic state tRNAs, in magenta and green), class 4B (new intermediates, in olive) and class 6 (hybrid state tRNAs, in gray), aligned with respect to the 70S ribosomes. (Figure adapted from [168], Copyright 2012 Proc. Natl. Acad. Sci. USA).

The rapid peptide transfer occurs after the accommodation of the incoming tRNA in the A site [149,150,151,152], leading to the formation of the so-called pre-translocational complex, ready to translocate the tRNA-mRNA complex by one codon in the 5' to 3' direction once unlocked [153,154]. First, the acceptor stems of the A and P tRNAs move with respect to the large subunit, generating the hybrid A/P and P/E configurations [17]. Kinetic data has shown that formation of the P/E hybrid configuration precedes the A/P state formation [31,41]. This tRNA reconfiguration is linked to the rotation of the 30S relative to the 50S along with the L1 stalk reconfiguration (first detected in EF-G bearing complexes, see [155,156]), and it is spontaneously formed in pre-translocational complexes as demonstrated by kinetic and FRET [29,30,34,39,157,158,159], and cryoEM studies of prokaryotic [160,161] as well as eukaryotic [162,163] complexes. X-ray data initially showed that the ribosome wraps intermediate rotated structures [164]. Later on, several intermediates, which include different tRNA configurations, have been described by cryoEM as well [165,166,167,168] (Figure 7), and the spatial and temporal relation between these transition state configurations and 30S subunit and L1 rearrangements computationally analyzed [61,62,63].

Complete translocation of the tRNAs from the A and P sites to the P and E sites respectively requires EF-G (or its eukaryotic counterpart eEF2, e.g., see [169,170]) and is coupled to the rotation of the head of the small subunit [171,172,173,174]. The factor, which undergoes a drastic structural rearrangement from a compact to an elongated conformation once bound to the ribosome [175], but before the hydrolysis of GTP [176], initiates mRNA-tRNA translocation by breaking the interactions established between the cognate codon-anticodon duplex and the decoding center [169,177,178,179]. Kinetic data have shown that the hydrolysis of GTP is very rapid and precedes translocation [31,180,181,182,183]. On the other hand, it has been demonstrated that EF-G can bind to the ribosome in either the classical or hybrid state to promote translocation [42,44,184], but favors the hybrid configuration [30,185,186]. It also has been shown that the final translocation of the tRNA-mRNA complex is coupled with the reverse rotation from the hybrid state to the classical state [187].

**Figure 8:**
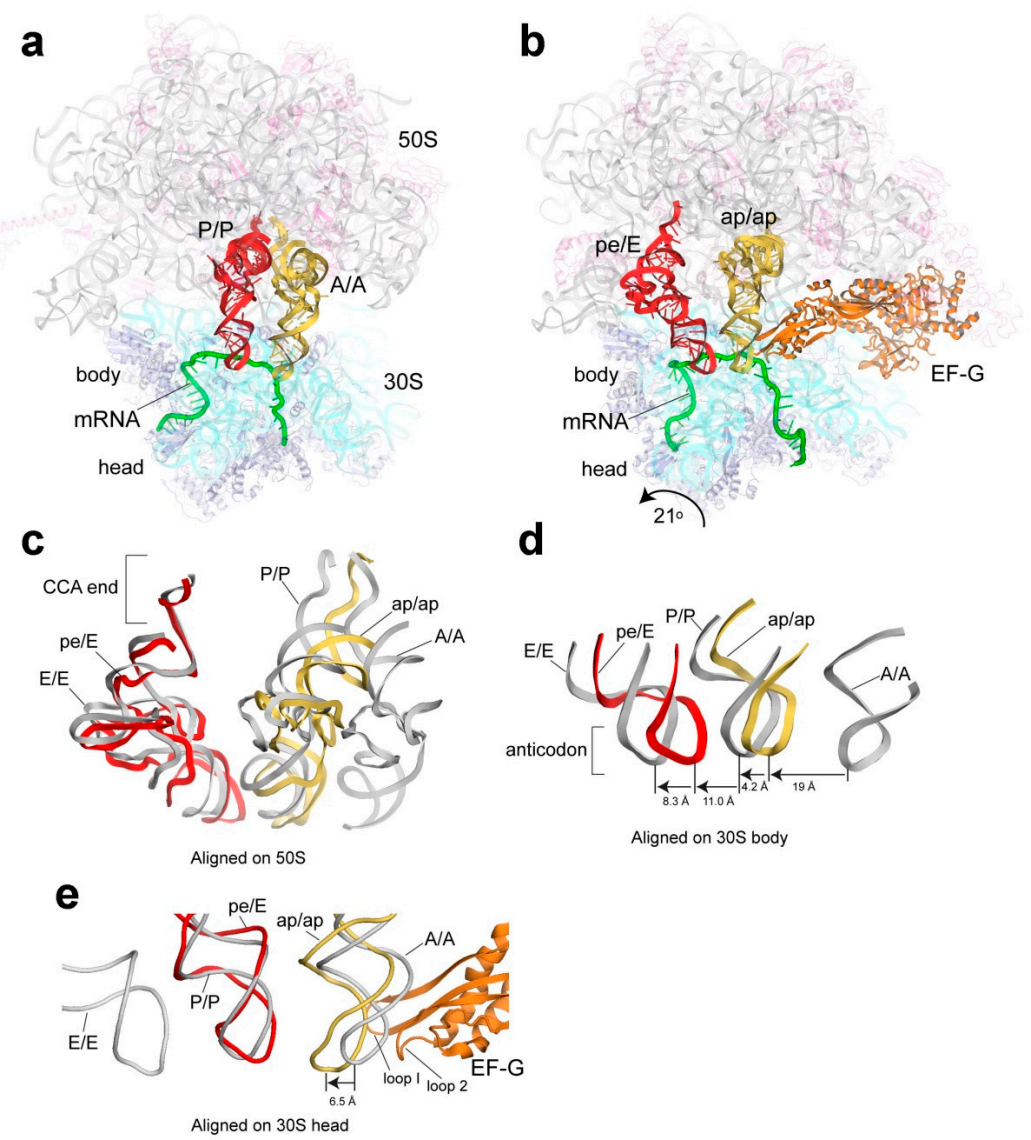
EF-G mediated mRNA-tRNA translocation intermediate. (**a**) Classic state pre-translocational complex (PDB 4V6F [188]); (**b**) Translocation intermediate in the presence of EF-G, bearing chimeric hybrid ap/ap and pe/E state tRNAs (lower-case letters imply that the corresponding tRNA is bound in a chimeric configuration, *i.e.*, halfway through the path from one canonical site to the next). Comparison of tRNA positions in classic and intermediate states after alignment of (**c**) 50S subunits; (**d**) 30S subunit bodies or (**e**) 30S subunit heads. (Figure adapted from [189], Copyright 2014 Am. Assoc. Adv. Sci.).

Recent high resolution structures of EF-G bound ribosomal complexes have shed light on the process [103,172,177,190,191,192,193,194]. One of the most relevant structures is the crystal structure of a *T. thermophilus* 70S ribosome containing EF-G and two tRNAs. The antibiotics neomycin and fusidic acid were used to block completion of translocation and to prevent the release of the elongation factor, respectively [189]. This is the closest structure of a true translocation intermediate described to date (also see [191,194] for lower resolution structures), and offers the first glimpse of the initial stages of the tRNA movement from the A site to the P site. The analysis of the structures showed that when compared to the classic state ribosome, the conformational changes of the small subunit (the head’s counterclockwise rotation of 21° coupled to the body’s 2.7° rotation relative to the 50S subunit) lead to the reconfiguration of the tRNA molecules (Figure 8). In the A site, the so-called ap/ap chimeric state tRNA configuration is observed: the ASL is bound between the A and P sites of the small subunit, whereas the acceptor stem is bound between the 50S subunit’s A and P sites, contacting both the A and P loops of the 23S rRNA that are involved in the peptide transfer reaction [195,196]. On the contrary, the chimeric pe/E state is formed in the P site, where the 30S head rotation places the ASL between the P and E sites via opening of the A790 gate [171], and the acceptor stem is located in the canonical E site of the large subunit. While the head rotation of the small subunit is sufficient to account for the observed displacement of the P-site tRNA, the same is not true in the case of the A-site tRNA. It was concluded that the movement of EF-G must have a major role in the partial translocation of the ASL of the A-site tRNA via interactions with the tip of domain IV [189].

## 4. The Stringent Response

Beyond the direct role in translation, tRNA molecules are involved in multiple metabolic and cellular routines [14,197,198]. Recent data also indicate that a mitochondrial tRNA is part of the architectural framework of the large mitochondrial ribosomal subunit (39S) itself, replacing the role of bacterial 5S rRNA as an organizing scaffold of the central protuberance region [199]. In the following section, we will focus on one important example of such a metabolic process, the induction of the stringent response by uncharged tRNAs, which serve as sensors for starvation and thus, are part of the regulation of global gene expression.

Living organisms face fast environmental changes upon which they have to adjust quickly and accurately in order to survive. Such important signaling pathways exist in all kingdoms of life, those related to modified nucleotides being the more prominent ones [200]. The bacterial stringent response, first identified due to the inability of certain *E. coli* mutants to reduce the amount of RNA synthesis in starvation conditions [201], is triggered during amino acid starvation (nutritional stress) and uses the alarmone (p)ppGpp (a hyper-phosphorilated version of GDP or GTP) to modulate a wide range of genes via transcription repression [202,203,204]. The factor responsible for sensing and producing the effective response, *i.e.*, the synthesis of the signaling molecule (p)ppGpp, is a ribosome activated enzyme termed RelA. Decades of biochemical and genetic studies have provided a detailed picture on how and when (p)ppGpp is produced in the cell and the stringent response activated. When bacterial cells are growing in exponential phase, only ~15% of all cellular tRNAs are deacylated [205], but most of them are engaged in regular cellular processes or ready to be re-aminoacylated. On the contrary, when the environmental conditions suffer a drastic change and there is scarcity of free amino acids, the concentration of non-acylated-tRNAs increases drastically, being able to interfere with the translation cycle by competing with the EF-Tu-GTP-aa-tRNA^aa^ ternary complexes to occupy the vacant A site of the ribosome. In this scenario, the peptide transfer from the tRNA in the P site to the tRNA on the A site cannot be performed and the ribosomes are stalled with cognate, non-acylated tRNA on the A site. This is the molecular event sensed by RelA, which binds to the stalled ribosomes to catalyze the transfer of the β and γ phosphates from ATP to GTP or GDP [206].

**Figure 9:**
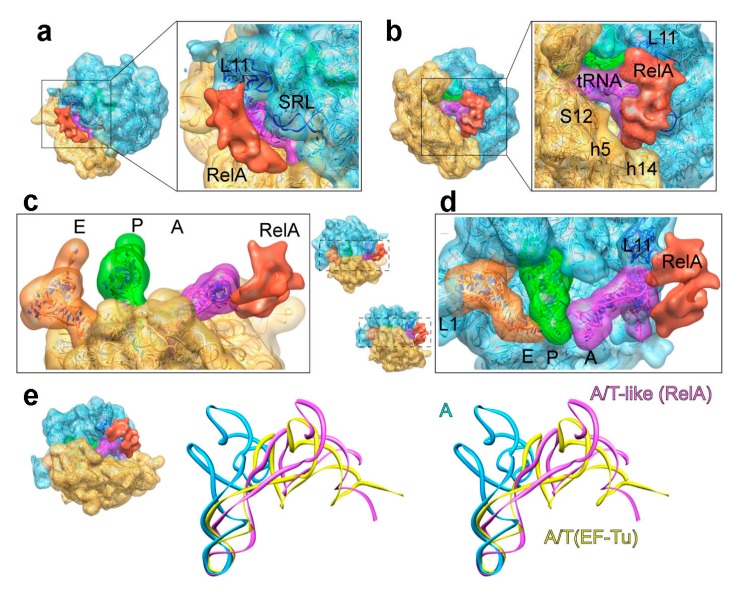
Sensing amino acid starvation. (**a**–**d**) 3D cryo-EM structure of the 70S–RelA complex. The molecular structures fitted to the density are shown in ribbons; (**e**) Stereo-view of the superposition of the A/T state tRNA in the presence of EF-Tu (PDB 4V5G [129]), the classic state A/A tRNA (PDB 4V5D [133]) and the A/T-like structure in the presence of RelA. (Figure adapted from [207], Copyright 2013 John Wiley & Sons).

A cryo-EM structure of the stringent factor RelA bound to translating but stalled ribosomes with cognate, deacylated tRNA species in the ribosomal A site was recently determined for the first time [207]. The structures revealed that RelA binds the large ribosomal subunit via L11 and the 30S subunit though interactions established with the shoulder domain of the 16S rRNA. Moreover, the structure showed that the deacylated tRNA is distorted into an A/T like state to interact with RelA, a configuration that enables the recognition of the deacylation state of the tRNA species as it exposes its 3' end. This pseudo A/T conformation is reminiscent of the one induced by the ternary complex with acylated-tRNAs during the incorporation of cognate tRNA species to the A site in a standard elongation cycle (Figure 9).

The novel conformation of the tRNA allows the proposition of a mechanism by which RelA might be activated, as it might serve as a scaffold to nucleate the catalytic 3D conformation of the factor. In a first step, the initial binding of the stringent factor to the stalled ribosome proceeds through the shoulder region of the small subunit, likely via the *C*-terminal region of RelA, which represents the regulatory and ribosome-binding domain [208,209]. This assignment based on (a) the binding of RelA to the ribosome is not dependent on the presence of L11 (as opposite to ppGpp production), as RelA can bind ΔL11 ribosomes [210]; and (b) no direct interaction is observed between the *C*-terminal ribosome-binding domain of RelA and L11 [209]. Next, RelA samples the A site of the ribosome through its *C*-terminal domain. The ability to contact the CCA end of a non-acylated-tRNA that has bounced backwards after non-being able to be successfully stabilized on the P site due to the absence of peptidyl transfer, allows a long lasting interaction, which results in a mutual stabilization. Reports show interactions between RelA and tRNA via the *C*-terminal region in other organisms [211,212,213], interactions that might allow the sampling of the 3' end of the oscillating tRNA. In a final step, due to the interaction of the elbow region of the tRNA with the L11-23S part, this GAC (GTPase associated center) region becomes more ordered and settles L11 to interact with the *N*-terminal fragment of RelA, the one which shows the associated ppGpp synthetase activity [214]. Again, the flexibility of the tRNA seems to be central for an efficient survey by stringent factor RelA of the charged state of tRNAs, and hence, the level of amino acids in the cell.

## 5. Concluding Remarks

In this review, we summarized the advances in the exploration of structural flexibility and dynamics of tRNA molecules during initiation, decoding and translocation stages of the protein synthesis cycle, most of which have only come to light in the past few years. These data emphasize the fact that the tRNAs do not traverse the ribosome’s inter-subunit space as rigid entities, instead, they show remarkable plasticity. Also highlighted was the distorted state of the deacylated tRNA during starvation signaling, a configuration that enables the recognition of the deacylation state of the tRNA species by the stringent response factor RelA. Although not mentioned throughout the text, but also worth mentioning are the initiator tRNA structures described during IRES-mediated initiation (e.g., [215,216]) or the hybrid, intermediate P-site tRNA configurations adopted during termination (e.g., [217]), recycling (e.g., [218]) or trans-translation related events (e.g., [219]). Some of the snapshots we focused on resemble the conformations adopted during simulations of tRNA molecules, but with the inherent structural constraints related to the binding at the corresponding interface. They also mimic some of the configurations visited during the computational simulations of entire ribosomal complexes. However, it is clear that the solved structures only represent a small fraction of the existing, relevant conformational states, as the highlighted conformations are just snapshots that were trapped in one of the local minima of the free energy landscape [220,221,222].

It is worth noting that until very recently, the only source of high resolution structures of ribosomal complexes was X-ray, both for prokaryotic [115] and eukaryotic [223,224,225,226] systems, whereas the lower resolution cryoEM structures were limited to describe conformational states and analyze domain movements/rearrangements in most of the cases. Right now however, cryoEM is transforming the field due to recent technical developments, which have enabled routine high-resolution, leading to a revolution in structural biology and in the ribosome field in particular [227,228]. Another accomplishment includes the use and development of time resolved methodology to capture short-lived intermediates [229]. Fully embracing the notion that the ribosome acts as a Brownian machine [230,231], the next step in the revolution is the development and use of new, unbiased tools to characterize the free-energy landscape and the continuous trajectories of the ribosome [232], without presumptions about the number of discrete subclasses that must be generated (as is the case with maximum likelihood methods). This will allow a further description of tRNA dynamics in the near future, as it will allow the classification of molecules such as ribosomes, which exhibit a continuum of conformational changes, and therefore, a more accurate comparison with the conformational trajectories, even the discrete sub-states proposed during the computational modeling and simulations.
